# A focus group study of enteric disease case investigation: successful techniques utilized and barriers experienced from the perspective of expert disease investigators

**DOI:** 10.1186/1471-2458-14-1302

**Published:** 2014-12-18

**Authors:** Stanley Ing, Christina Lee, Dean Middleton, Rachel D Savage, Stephen Moore, Doug Sider

**Affiliations:** Dalla Lana School of Public Health, University of Toronto, 155 College Street, Health Sciences Building, 6th Floor, Toronto, Ontario M5T 3M7 Canada; Public Health Ontario, 480 University Avenue, Toronto, M5G 1V2 Canada; Department of Clinical Epidemiology & Biostatistics, McMaster University, 1280 Main Street West, Hamilton, Ontario L8S 4K1 Canada

**Keywords:** Enteric, Case investigation, Telephone interview, Interview techniques, Focus group, Foodborne illness

## Abstract

**Background:**

In Ontario, Canada, enteric case investigators perform a number of functions when conducting telephone interviews including providing health education, collecting data for regulatory purposes ultimately to prevent further illness, enforcement, illness source attribution and outbreak detection. Information collected must be of high quality as it may be used to inform decisions about public health actions that could have significant consequences such as excluding a person from work, recalling a food item that is deemed to be a health hazard, and/or litigations. The purpose of this study was to describe, from the perspectives of expert investigators, barriers experienced and the techniques used to overcome these barriers during investigation of enteric disease cases.

**Methods:**

Twenty eight expert enteric investigators participated in one of four focus groups via teleconference. Expert investigators were identified based on their ability to 1) consistently obtain high quality data from cases 2) achieve a high rate of completion of case investigation questionnaires, 3) identify the most likely source of the disease-causing agent, and 4) identify any possible links between cases. Qualitative data analysis was used to identify themes pertaining to successful techniques used and barriers experienced in interviewing enteric cases.

**Results:**

Numerous barriers and strategies were identified under the following categories: case investigation preparation and case communication, establishing rapport, source identification, education to prevent disease transmission, exclusion, and linking cases. Unique challenges experienced by interviewers were how to collect accurate exposure data and educate cases in the face of misconceptions about enteric illness, as well as how to address tensions created by their enforcement role. Various strategies were used by interviewers to build rapport and to enhance the quality of data collected.

**Conclusions:**

To our knowledge, this is the first study to examine the perspectives of expert enteric disease case investigators on successful interview techniques and barriers experienced during enteric case investigation. A number of recommendations could improve the process of enteric case investigation in the Ontario context which include formal training and development of resource materials pertaining to interviewing, standardized interviewing tools, strategies to address cultural and language barriers, and the implementation of the single interviewer approach.

**Electronic supplementary material:**

The online version of this article (doi:10.1186/1471-2458-14-1302) contains supplementary material, which is available to authorized users.

## Background

Enteric, or gastrointestinal, illnesses continue to be an important global public health issue [1]. In the province of Ontario, Canada, the number of reportable gastrointestinal illness cases for the years 2007 to 2009 was 10,746, 10,125, and 9,026, respectively. These cases included amebiasis, botulism, campylobacteriosis, cryptosporidiosis, cyclosporiasis, giardiasis, hepatitis A, listeriosis, paratyphoid fever, salmonellosis, shigellosis, typhoid fever, illness due to verotoxin-producing *Escherichia coli*, and yersiniosis [2]. With respect to modes of transmission, foodborne transmission was assessed to make up 54% of domestically acquired enteric illnesses. Animal contact, person-to-person, water and "other" modes of transmission made up the remainder [2]. The estimated annual cost of foodborne illness in Canada is $3.7 billion Canadian and includes costs attributed to health care services, lost productivity and missed paid employment [3, 4]. Given the significant burden and economic costs of foodborne illness in Ontario and Canada, the prevention and control of foodborne, and all enteric, illness continues to be a priority.

In Ontario, investigation of reportable enteric disease cases is mandated through legislation described in the Methods section. In general, cases are investigated for five reasons: 1) collecting data elements required under the *Health Protection and Promotion Act*, Regulation 569, Reports [5]; 2) case and contact management; 3) providing education to prevent further transmission of disease; 4) determining the source, or possible sources if the definitive source is not identified, of the disease-causing agent; and 5) identifying potential outbreaks through linking cases who share a common source of illness [6, 7]. The information obtained for these purposes is collected predominantly through interviewing cases via telephone. The information may also be used further for making public health decisions that may have significant consequences such as excluding a person from work, recalling a food item that is deemed to be a health hazard, and/or closing a business. Thus, it is critical that high quality information is collected to avoid placing an unwarranted burden on Ontario residents. High quality information, as it pertains to enteric case follow-up, refers to the ascertainment of complete and accurate information (e.g., exposures and risk factors, travel history, occupation, etc.) from each enteric case. Occasionally, the evidence supporting public health decisions is required to withstand legal scrutiny during litigation.

Interviewing enteric disease cases differs from other types of interviews such as opinion polls, social science interviews, clinical trial interviews, or self-administered questionnaires. Some of the unique and often challenging aspects of conducting enteric case interviews include:

1. Cases being interviewed are not volunteers; they did not volunteer to become ill,
2. Cases are not compensated for the time and disruption associated with case investigations,
3. Some of the roles that investigators play may be conflicting; for example, investigators have to elicit information from the case, be empathetic to the case’s illness, and act as an enforcer if the case has to be excluded from work,
4. Probing for information where the person may be perceived as being at fault for causing illness such as inadequate sanitary practices, food handling failures, and unsafe sexual practices,
5. The requirement to assimilate a large amount of unstructured information from the case, and from contextual and situational information pertaining to the disease that will ultimately be used to prevent further illness, and
6. Interviewers do not get to choose their cases and have little information about their case prior to contacting them. For example cases could be infants, could present unanticipated language or cultural barriers, could be hospitalized (and unable to speak), or could be next of kin and in the process of mourning the loss of a loved one.

Thus, while there are plenty of scientific, evidence-based best practices available for opinion polls, self-administered questionnaires, etc., enteric case interviewing can only draw on the learnings from these findings to a limited extent. The unique and challenging aspects of enteric case interviewing underlines the critical role that interviewers play in the public health system and the tremendous skill required to perform the role.

A number of jurisdictions in the United States and other countries have documented techniques which aid investigators when conducting case investigation, however, there is a dearth of empirical evidence examining successful techniques used for enteric case investigations [8–15]. A number of techniques have been described as facilitating successful interviews with enteric cases including building rapport with cases, ensuring confidentiality of information provided to public health, using effective communication skills, remaining objective, providing sympathy, and using open-ended and close-ended questions [10, 13–15].

To gain further insight into the practices and techniques used by investigators, focus groups were conducted. The purpose of this study was to describe, from the perspectives of expert investigators, techniques used and barriers experienced as well as the techniques to address these barriers, during investigation of enteric disease cases. In addition, perspectives were sought from investigators on the methods used to identify potential outbreaks through linking two or more cases with a common source. Investigators were also asked to provide recommendations for improving the overall process of enteric case interviewing in Ontario.

## Methods

**Figure 1:**
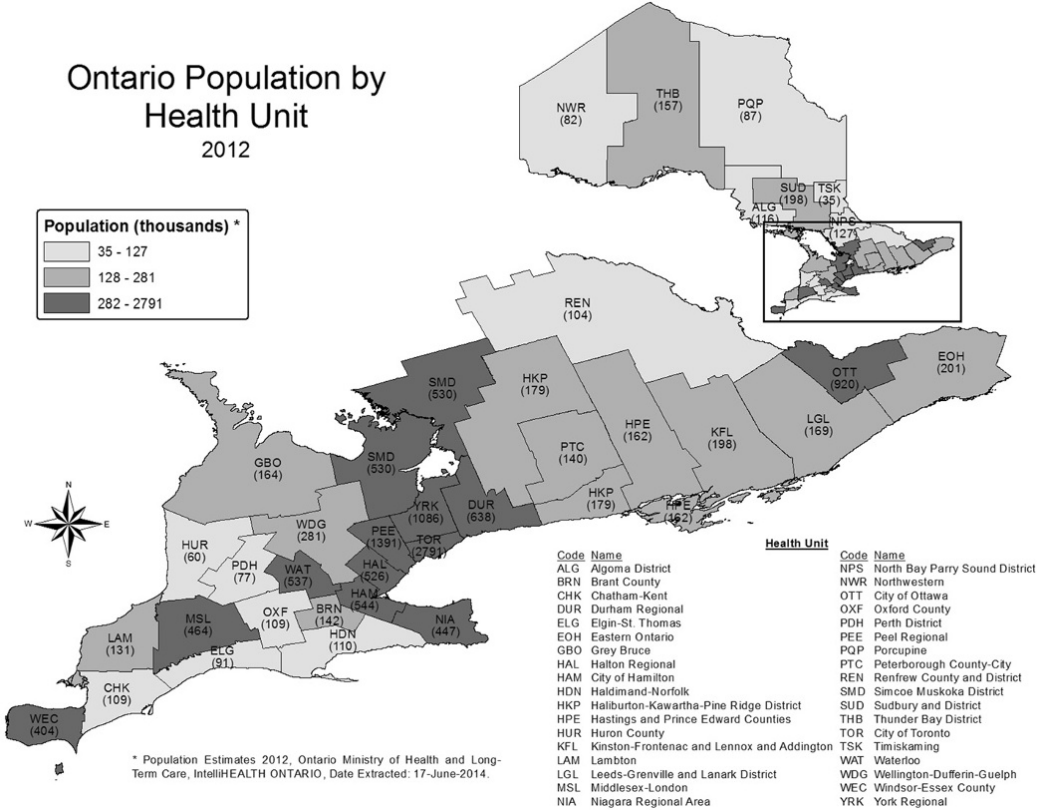
**Ontario, by public health units and population, 2012.**

Ontario is a province in Canada with an estimated population of 13,762,000 in 2014 [16]. The geographic boundaries of the 36 autonomous public health unit jurisdictions in Ontario as well as the respective populations of the health units is shown in Figure 1. For the purposes of descriptive findings in this study, the health units were categorized by peer groups. A peer group is defined as a cluster of health units with similar social and economic factors which use 2007 health unit boundaries and 2001 census data [17].

In Ontario, the Ontario Public Health Standards published by the Ministry of Health and Long-Term Care, under the authority of the *Health Protection and Promotion Act* [5], specifies the mandatory health programs and services provided to the public by the 36 public health units [18]. The Infectious Diseases and Food Safety Protocols, under the Ontario Public Health Standards, provide direction to health units with respect to food safety, the prevention and management of infectious diseases, as well as the identification and management of outbreaks [6, 19]. A number of individuals who include physicians, hospital administrators, laboratory operators, school principals and superintendents of institutions, have a legal requirement to report to the Medical Officer of Health, the lead of the respective health unit with respect to reportable diseases, who in turn has a legislated obligation to report the information to the Ministry of Health and Long-Term Care. Public health unit staff must then perform investigation of enteric reportable disease cases for the purposes of:

1. Collecting data elements required under the *Health Protection and Promotion Act*, Regulation 569 [5],
2. Case and contact management,
3. Providing education to prevent further transmission of disease,
4. Determining the source/potential sources of the illness, and
5. Identifying potential outbreaks through linking cases with a common source [6, 7].

These five requirements are collectively identified in the following text as "legislated requirements". Enteric case investigation is usually conducted by public health inspectors (known as environmental health officers in some jurisdictions) and public health nurses. Data from case investigations are reported to Public Health Ontario, as an agent of the Ministry of Health and Long-Term Care, through a dynamic, web-based application for reporting and managing reportable disease information.

### Study design and ethical approval

A purposive sampling approach was used to recruit expert investigators. Information about the study and a nomination form was sent to each of the 36 health units by e-mail to invite their participation. Investigators who had at least two years of experience interviewing enteric disease cases in a health unit since 2009 were eligible to be nominated. There was not a limit to the number of expert investigators that a health unit could nominate. To help ensure that nominees were recognized as expert investigators within their health unit, supervisors, managers and/or peers were responsible for nominating the expert investigators. A number of factors guided the decisions in nominating the investigators within their health unit including:

1. Consistently obtaining high quality data from cases by enhancing engagement and recall of the potential sources of illness,
2. Achieving a high rate of completion of case investigation questionnaires,
3. Identifying the most likely source, or potential source if the definitive source was not identified, of the disease-causing agent, and
4. Identifying any links between cases that may be caused by a common source.

The nomination forms were sent to the study team by a supervisory staff member with the names of the nominated expert investigators. Each potential participant was contacted and provided with a description of the purpose of the study and consent to participate in the study by the participant was obtained. This project was assessed through the Public Health Ontario Ethics Review process and was granted approval for a period of one year commencing January 14, 2014.

### Data collection and analysis

The proposed number of expert investigators in each focus group was six to eight as this range would be sufficient for providing a variety of perspectives [20]. It was expected that three to four focus groups would be required to answer the research questions.

Focus groups continued to be conducted until a clear pattern emerged in the analysis and theme saturation was obtained [21]. A topic guide outlining broad, open-ended questions was used to guide the focus groups (see Additional file 1). Examples of questions included "What do you do to establish and maintain rapport with cases?" and "What are the barriers in establishing and maintaining rapport with cases?". While the guide provided structure and direction to the focus groups, questions were adapted to attend to participant responses. The topic guide was piloted prior to commencement of the focus groups with two colleagues who had experience with enteric case investigation, and feedback was incorporated into the final topic guide. Focus groups were conducted via teleconference in the month of February, 2014 and lasted 2 to 2.5 hours each. A focus group facilitator moderated the focus groups while two other research team members supported the facilitator by taking notes during the focus group to capture preliminary themes. All focus groups were audio-recorded using a digital recorder and the participant’s comments were transcribed verbatim.

**Figure 2:**
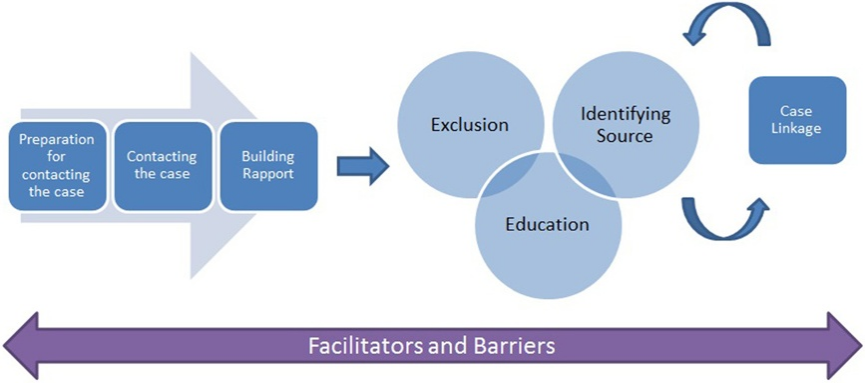
**Framework for enteric case investigation.** Framework depicting the sequence of an enteric case investigation used to group data based on the topic guide questions.

Transcripts were subjected to concept saturation and theme generation analysis using NVivo (QSR International Pty Ltd. Version 10, 2012) by a graduate research assistant trained in qualitative methods and analysis. Thematic analysis using a deductive/theoretical approach was used to analyze the transcripts for the purposes of identifying, analyzing and reporting patterns within the data [22]. A framework was developed *a priori*, based on experience, depicting the sequence of an enteric case investigation in order to group data based on the topic guide questions (Figure 2). The initial components of the framework include preparation for contacting the case, contacting the case for the investigation, and building rapport. Education is provided to the case in regard to avoiding becoming infected again and preventing further transmission of the pathogen. Efforts are made to identify the most likely source/potential sources of infection. Where required, investigators take action to exclude cases from high risk settings. Exclusion refers to excluding a case from working in a food premises, working in a healthcare setting, or working in or attending a childcare setting because of the risk of transmitting the pathogen to others. These settings are referred to as high risk settings. The duration of exclusion varies by pathogen. Further, stool clearance is required for cases of typhoid fever, paratyphoid fever, shigellosis and verotoxin-producing *Escherichia coli*. Stool clearance requires a certain number of stool samples to test negative for the pathogen before returning to the food premises, healthcare setting or childcare. Efforts to link cases with a common source may then be undertaken after the interview. Themes were generated from the identified components of the framework.

The analysis was guided by the proposed phases of thematic analysis outlined by Braun and Clarke which included: 1) data familiarization, 2) generation of initial codes, 3) searching for themes, 4) reviewing themes, and 5) defining and naming themes [22]. Qualitative rigor was achieved through inter-rater reliability in which 25% of the raw data was reviewed and coded by three independent reviewers and member participant checking by presenting the study themes to focus group participants via webinar and requesting feedback. Consensus agreement on the themes was achieved between the reviewers via in-person discussion prior to further coding. Participant's quotations used in this manuscript underwent minor edits to improve readability. The point of the comment was always maintained. Square brackets were used to indicate edits made to the quotations.

## Results

A total of 28 expert enteric case investigators from 18 health units across Ontario were nominated. All 28 investigators participated in one of four focus groups via teleconference. Of the 28 participants, 20 were public health inspectors, seven were public health nurses, and one was a public health nurse/epidemiologist. Participants’ years of experience investigating enteric diseases ranged from 2 to 25 years (average: 8.9, median: 7.0). The 18 health units represented five of the six peer groups in Ontario, which included urban/rural mix, urban centres, sparsely populated urban–rural mix, mainly rural, and metro centre. Compared to the 18 health units that did not have interviewers in the focus groups, the 18 health units with staff participating in the focus groups represented more health units classified as urban centres and fewer health units classified as mainly rural. The participating health units served larger populations compared to the non-participating health units. The participating health units serve approximately 75% of Ontario’s population.

Although clear themes emerged within each component of the framework, participants reported using a variety of techniques or strategies to optimize the interview process and address specific barriers. The themes that emerged from the data are presented under their respective framework headings below and where possible, examples are provided to highlight the diversity in approaches or strategies used by the participants.

### Enteric case investigation preparation and case communication

When participants were asked to discuss their methods for contacting cases, two themes emerged from the discussions: understanding contextual information, and barriers and strategies to contacting cases.

#### Understanding contextual information

For some participants, a review of the characteristics of the enteric disease prior to case investigation provided investigators with a quick "refresher" which enabled them to be mindful of the incubation period, common sources of infection, modes of transmission and control measures when communicating with the case. Participants also indicated that this familiarity with the pathogen characteristics assisted in the ascertainment of the legislated requirements. In addition, participants identified the importance of being familiar with the demographics of the case. Participants often described that demographic information found on the laboratory report was a rich source of information. This information included gender, age, residential home address and contact information for the case, as well as the setting where the specimen was collected. Some participants stated that they sometimes use the case’s last name as a method to identify their ethnicity. By doing so, participants were able to possibly identify common cultural foods that were consumed within the home. For example, one participant commented: "*I take a look at the person*’*s name to get a gauge of their ethnic background*…*you have an idea of how they might prepare food*, *where they might eat*…*there are lots of people who may wash their chicken before they cook it. Some people sample raw meat before they actually cook it*". Furthermore, participants also reported how examining the case’s last name could assist in predicting if language may be a potential issue during the interview, as illustrated by one participant: "…*[if] I see the name of the person and the ethnicity of that person*, *I can ask somebody who probably speaks the language just to leave a message in their native language*". Lastly, maintaining situational awareness was cited as an important activity prior to case investigation. Expert investigators made themselves aware of current provincial or local food recalls, outbreaks, or enhanced surveillance directives. Enhanced surveillance directives are issued by provincial authorities in order to assist with the provincial investigation of urgent situations and/or to obtain data required for timely surveillance. When an enhanced surveillance directive is issued, the requested data become a priority for reporting to the province. In some situations, this enabled participants to establish possible linkages of cases to outbreaks.

#### Barriers and strategies to making contact with the case

Upon receiving the laboratory report, a number of participants cited challenges with respect to missing or incorrect case information. For example, one participant described: "*some of our lab slips*; *we won*’*t even have the proper address on it or the proper phone number to call someone*". In response, participants often had to investigate further to obtain this information; this could include contacting the laboratory, the case’s physician, using various websites such as 411.ca (a search engine for finding people or businesses in Canada), or using other internet search engines.

Participants also cited barriers to initiating contact with cases due to health unit policies in place for the purposes of ensuring confidentiality. For example, one participant indicated: "*[when] our health unit calls*, *it shows [a] blocked number*, *like they don*’*t want our number showing because obviously, it might be someone from clinical or sexual health… if it was someone else answering the phone…*". While these policies are implemented to ensure the confidentiality of cases, blocking telephone numbers can also deter cases from picking up the phone. In response, some participants had cited using unlisted numbers such as their personal phones or on-call cell phones for case investigation.

In the event that the case did not answer the phone, participants would usually leave a voice mail. For a majority of participants, it was stated that certain elements would be included in the voice mail; their name, health unit, contact information and office hours. In addition, participants also described that they would request alternative contact numbers for the case or more appropriate times to contact the case. Participants emphasized that no mention of their department or the case’s personal health information should be included in the voice mail for privacy reasons. With the evolving use of different communication technologies, one participant provided the example of using text messages to communicate with difficult-to-reach populations: "*With students and people who don’t [*check*] their voicemail or answering machines or smart phones…I’ve started texting and I find that it gets a response*". Upon reaching the case, participants often discussed that they would provide the case with the opportunity to choose a time that was convenient for them to do the interview, as described by one participant: "*I would call them and let them know that I need to do a quick interview, and I would put the onus on them to tell me when they had time to actually speak to me…I think keeping them on the phone is easier if it’s on their time schedule*". A majority of participants would often ask at the beginning of their interview if the case had the time to complete the interview.

### Establishing rapport

Once contact had been initiated with the case, participants described a number of techniques and barriers to establishing rapport with cases. Two themes emerged from the discussions: easing case anxiety, and the need to be respectful of the language and cultural identity of the case.

#### Easing case anxiety

Participants often explained that to build rapport with cases for the purposes of eliciting information, investigators must ensure that cases are comfortable during the interview process. Participants had stated numerous methods which assisted in the process of establishing rapport. Upon initiating contact with cases, a majority of participants would promptly explain the purpose of the interview and the role of public health. Some participants indicated that in some situations, cases may feel a bit anxious upon initial contact. For example, one participant described how defining the role of public health can help to motivate case participation: "*Not everyone is familiar with public health and [if I] explain what our role is, that we just want to prevent illness in other people, [it] can sometimes help*". In addition, a number of participants also indicated that during the investigation, confidentiality would be thoroughly explained with the case. To further decrease case anxiety, a majority of participants emphasized the necessity of being empathetic, to allow cases to tell their story, and to conduct the interview in a conversational style. For example, one participant stated: "*I also try to start with their symptoms first, so asking them questions about their symptoms sort of gives an opportunity to show a bit of empathy. I find when you try to talk about symptoms, people are really interested to tell you…how sick they were and how they think that they got sick. So I try to show that we’re empathetic towards that and give them a bit of time to give their own story before I move onto our agenda of questions that we want to get into*".

#### Identifying and being respectful of the language and cultural identity of the case

Although participants described techniques for establishing rapport, participants also cited a number of barriers. One of the main barriers participants identified was language. Participants who identified language barriers were from health units classified as urban centres. To overcome this barrier, many health units have access to third-party translation services; however, some participants identified difficulties in finding appropriate times for the case, the translator, and the investigator to connect. In addition, participants also reported that for situations where the case is not fluent in English, the phone is usually passed to another member of the household. For these scenarios, participants would request permission from the case through the household member to translate and disclose personal health information of the case. For example, one participant indicated: "*If they speak no English at all, then usually, like right off the bat, they’ll give the phone to someone that does speak English in their household and so hopefully, you can kind of get permission that way. It could be a bit difficult sometimes to get permission…we continue the interview with the person who could speak English. If English isn’t their first language but they can speak English, I just try to get through the interview with them, and for them, it’s important that you’re speaking very slowly and very clearly, and not using very advanced words".* Some health units have staff who speak a variety of languages. One participant reflected briefly on the differences in using translation services when compared with a staff member who can speak the case’s language: "*We have a lot of investigators that speak most of the languages that are in [name of health unit] and we find it a lot easier when speaking [with] somebody… they want somebody who is [a certain ethnicity]*". Culture may act as a barrier during the case investigation. This was illustrated by a female participant who stated: "*There’s some cultures where the interviewee has told me that sometimes only the male wants to talk to a male interpreter, they don’t want to talk to a female*". In addition to the complexities introduced by culture and how it could act as a barrier, participants also reported the anxiety felt by cases that identify as immigrants or refugees when speaking with public health. To address this barrier, participants would often need to convey to the case that they weren’t in trouble, as indicated by one participant: "*In terms of cultural barriers…being very sensitive…show an interest to learn about what it is they do in their culture, how they are preparing dishes, people do like to share…their traditions and cultural beliefs and we make very clear that it’s confidential, nobody is going to get in trouble, we just want a better understanding of what is being done and then again remind them of what the goal is …to prevent further transmission*". Lastly, participants described the difficulties in reaching certain populations such as religious communities. One participant illustrated the intricacies of conducting case interviews within a local religious community: "*Some orders, they don’t let their wife or family members talk to us so we would have an interpreter [from] the men of the house*". Another participant illustrated the resources required to conduct investigations in these communities and the respect public health must provide when communicating with cases: "*We don’t have a super large [certain religious] population but we certainly have pockets in our area and I know our health unit is looking to have more of a…liaison with that community and having contacts here at the health unit who have contacts with the different orders in that community, and so sometimes for specific investigations, we may go through our health unit contact to help us with investigations. … really we should be cognizant of their religion and privacy practices*".

### Source identification

When participants were asked to discuss their methods for identifying the most likely source/potential sources of illness, three themes emerged from the discussions: education as a means to counteract the public’s misconceptions of foodborne illness, taking a non-judgmental approach to soliciting sensitive information, and approaches to improving recall. An educational component was involved in many of these themes.

#### Education as a means to counteract the public’s misconceptions of foodborne illness

For a number of participants, the interview usually began with education in regard to pathogen characteristics such as common sources, how the pathogen was transmitted, and incubation period. Providing this information was used by some participants as a way to address cases’ misconceptions about the potential source of their illness. For example, one participant stated: "*When I do start my interview, I always start with the education first… people often tend to think that it’s the last thing that they ate that made them sick, so if you give the education first, then you explain the incubation period, then they realize that the last meal they ate in fact couldn’t be what made them ill, so I find it helps to start it off that way*". In addition, cases may also have a specific food item or food premise in mind which they believe had been the cause of their illness. For example, one participant described how this may act as a barrier to source identification: "*One barrier that we come across is when you phone someone up, and they know exactly where they got this from because they went to [fast food restaurant] this morning and that’s just for sure what happened, and they’re not [open to exploring] other possible risk factors*".

#### Taking a non-judgmental approach to soliciting sensitive information

Participants emphasized the difficulties of identifying how the case became ill when the most likely risk factor attributed to the illness was due to high-risk behaviours or personal practices such as poor hand hygiene or sexual practices. For example, one participant stated: "*You can look at the risk factors in iPHIS [Ontario’s web-based reporting system for reportable diseases] and they answer no, no, no, and at the end, you feel the hesitation in their voice especially when it’s sexual contact or intercourse*". In addition, participants explained that cases often viewed themselves as good hand washers, as illustrated by another participant "*So I’ve never spoken to anyone that’s ever admitted they weren’t a good hand washer, so not seeing what some of their personal practices [are] can be a barrier to identify the source*". When high-risk behaviours or personal practices are considered to be the most likely source of how the case had become ill, participants stated that it is essential to convey a non-judgmental approach during the interview. This attempts to ensure that the case is comfortable in disclosing sensitive information on behavioural risk factors.

#### Approaches to improving recall

It was widely cited by a majority of participants that poor case recall was the principal barrier for the identification of potential sources of infection. For example, one participant illustrated, "*The time that they had symptoms, going to the doctors, then getting the results, there could be a fair amount of time…so that’s a barrier…being able to remember … what they were eating*". In response to poor case recall, participants described using a number of tools and methods to prompt recall. All participants described using a case report form as a tool during the interviewing process. A case report form typically contains questions on the common risk factors for the pathogen. The inventory of risk factors assists the investigator with systematically identifying the risk factors to which the case may have been exposed. Further, querying the case assisted with prompting the case’s recall in regard to the risk factors. Some participants cited that they often pose additional questions to those on the case report form, including shopping habits, product brands, and grocery store names, to assist with identifying the source of illness. The use of open and closed ended questions were viewed as complementary. One participant reported: "*Sometimes asking the closed-ended questions also helps to jog memory because somebody may not think…, you ask them specifically about cantaloupe, or lettuce, or something like that and it can help to generate some more conversation, but the open-ended questions are definitely helpful, again for storytelling and the building of rapport*". Many participants indicated using a calendar to prompt recall during their interview, as stated by one participant: "*I have always used the calendar trick where I would ask them to have a calendar in front of them or use some sort of event like a family barbeque, a wedding, or something just to relate them back to where they may have been, which helps trigger what they may have consumed*". Some participants would also ask the case if they had a loyalty card for their grocery store, debit transactions statements, or purchase receipts to identify possible food sources and to assist in the case’s recall. In instances where no risk factors are implicated, participants would often investigate personal behaviours. One participant described: "*I also try to go around behaviours as well, so if we can’t really identify anything, if there have been no special events or if there has been no eating out at any restaurants, or anything that we could identify… so asking things about cutting boards and utensils, and those sort of things to get around cross-contamination".*

### Education to prevent disease transmission

When participants were asked to discuss their educational strategies to prevent further disease transmission, three themes emerged from the discussions: using the interview as an educational opportunity to prevent future infections, preventing household disease transmission, and providing education to cases who are non-accepting of the probable cause of their illness.

#### The interview as an educational opportunity to prevent future infections

For most participants, once the most likely source/potential sources of infection had been identified, participants would proceed with providing targeted education with respect to preventing further transmission. A number of participants cited providing continuous education throughout the interview. One participant indicated: "*I would say at the beginning, the education is like an overview so you’re providing them with kind of like your summary of a fact sheet…letting them know about the disease…having that sense of how they might have been exposed will let you focus on that point of education that you want to give to them*". In addition, some participants also explained that throughout the interview process, they would explain the rationale behind the questions that they would be asking, as described by one participant: "*I’ll give them information on the organism just so they have some background information but as I go through, for example, for Salmonella, we ask if they’ve re-heated any frozen poultry products in the microwave, and I’ll say ‘we ask you this because it can create cold spots’…there might still be some raw stuff… I build it in as I go and then also at the end, I talk about preventing transmission like hand hygiene, if you cook poultry to use a thermometer, that kind of thing*". At the end of the interview, some participants would also offer a fact sheet to the case.

#### Preventing household disease transmission

Participants described taking the time to ask about household members that were also ill. Additional education efforts may occur in the form of identifying possible household transmission routes, as indicated by one participant: "*Looking at what contacts are in a household and … making education specific so if it’s a child … are they bathed with other siblings…trying to get to some of those … finer details*". Participants often cited providing education on cleaning practices within the home, as described by one participant: "*We go through how they’re cleaning their washrooms. I talk to them about the different disinfectants and if they’re using a disinfectant".* Some participants asked if a specific food, identified as the most likely source of infection, was still within the home. In these cases, public health laboratories could test the food product for the presence of the food pathogens or toxins, or instruct the case to discard the food product to prevent infection.

#### Educating cases who are non-accepting of the probable cause of their illness

Based on the situation, some participants emphasized the challenge of providing education on certain high-risk behaviours and cultural practices. One participant indicated: "*It gets more difficult especially when dealing with something that could be anal-oral contact, that’s the hardest part, and trying to relate to that with somebody who culturally wouldn’t be discussing that over the phone with a stranger*". Furthermore, some participants also described how cultural beliefs can impede the uptake of health education as illustrated by another participant: "*… somebody with amebiasis and in their culture, they tend not to use toilet paper but a toilet bidet system to clean themselves and this [is]…difficult to discuss. A lot of the time, they don’t want to discuss toilet habits ‘cause it’s not discussed at all, and…if you have amebiasis, it could be spread sexually through anal-oral contact, it’s sort of a taboo, and then…they will hang up at that point*".

Some participants described culture as a barrier in providing education to travel-related cases. Cases would often indicate that the foods consumed while travelling to their home country are of the norm for their culture. It is certainly recognized that any food that is contaminated with a pathogen could result in illness. Nonetheless, this often creates difficulty for the investigator as cases are not open to listening to the risks associated with the foods that they had consumed while travelling abroad.

Misconceptions about safe food handling practices occurring within the home were a common experience among participants. For example, one participant discussed food handling practices within the home as it pertains to cultural food items: "*Food handling practices within the home, which…seemingly goes against what we could consider safe food handling… they don’t see what they are currently doing as an issue or against anything that could cause an ill-perceived health issue because they’ve been doing it for so long*".

To address the barriers in providing education to cases, participants often cited using their interpersonal skills such as being sensitive, neutral, and non-judgmental when speaking with cases. One participant explained the approach taken when educating a participant on risks associated with the consumption of unpasteurized milk: "*We try to be very non-judgmental but explain the risks because I find, especially with unpasteurized milk, they feel very strongly about it … that if it’s someone that says yes to that, they’re sort of anticipating an argument. So we usually just say ‘Well, just an FYI, our recommendation is not to [consume unpasteurized milk] for the following reasons’, but… I think it’s in your delivery and not to sound like you’re telling them what to do, or being judgmental about it even though obviously you’re trying to direct them to doing something less risky".* One participant indicated using an approach to education via a hypothetical case or example as a way to address education in a sensitive manner: "*We’ll allude to members of the public but not [the case] and so hopefully they get some of the information, so it’s kind of a more passive approach to… relaying the information as an FYI, I heard this…happened and that’s the only way because some people take huge offence. I had a man who repeatedly shouted at me that’s he’s not gay and he sort of misunderstood that I was trying to infer that it’s just anal-oral contact so… you just lose the person totally*".

### Exclusion

When participants were asked to discuss their strategies to prevent further disease transmission through exclusion (i.e., excluding a case from working in a food premise, working in a healthcare setting, or working in or attending a childcare setting because of the risk of transmitting the pathogen to others), a theme related to tensions between identification, education and enforcement emerged.

#### Tensions between identification, education, and enforcement

In terms of preventing disease transmission for the purposes of exclusion, participants described the need to identify whether the case worked in a food premises or healthcare setting, or worked in or attended a child care setting. Where it was identified that a case was working in or attending a high-risk setting, participants described the exclusion process to the case for the purposes of the legislated requirements. Participants also provided education on the rationale for the decision for exclusion. One participant indicated: "*We always follow with verbal education…we discuss the sources of the illness, hand hygiene, cooking temperatures, safe food handling techniques*". Some participants described that for some excluded cases, stool clearance was required.

Participants identified a number of barriers when excluding cases. For example, one participant described the experience: "*When you’re speaking with someone basically paid by the hour, where they don’t get benefits, they don’t get sick time, they’re very…reluctant to stay home*". Individuals may also fear losing their jobs. They may change their story if they realize that they may be excluded, as illustrated by one participant: "*They may have told you that they’re a food handler but they may start telling you ‘Well actually, I don’t touch the food, I don’t prepare the food’*". Some participants indicated that they try to identify occupational status closer to the end of the interview when some rapport has been built. Asking for the case’s occupation at the beginning of the interview may cause the conversation to deteriorate and as a result, the case may not provide the investigator with any further information. To ensure cases are excluding themselves from work, participants described a number of techniques; however, there was variability with respect to how successful each technique was to ensure that cases were actually complying. Some participants informed employers that a case would not be allowed to work. In these situations, they would not provide any details with respect to the case’s illness for privacy reasons. Health inspectors may follow-up with a food premise to observe if the excluded case is complying. Participants often describe that ultimately, they rely on the case being honest in compliance. However, one participant indicated: "*We have gone down the legal route… saying that we will take legal action if they will not stay off work but we rarely have to go down that route. Usually, we just explain that we can take legal action and that’s enough to have them excluded*".

### Linking cases

When participants were asked to discuss their methods for linking cases who share a common cause of illness, communication and health unit processes emerged as themes.

#### Communication

A majority of participants described the importance of communication in linking cases. Participants would often collaborate with data entry staff, other enteric disease investigators within their health unit and with other health units, public health inspectors in the food safety program, and provincial public health agencies. Occasionally, enteric disease investigators in other health units may be contacted. For example, participants described communicating with health inspectors if it appeared that a food premise was implicated as the source of the outbreak. A linkage between the source of the infection and cases may be established if the health inspector received numerous notifications from different enteric case investigators. Further, participants often cited communicating with other investigators within their health unit to identify if cases were linked. Some expressed that given their small health unit or team structure, most of the investigators sit in close proximity of each other, which facilitates discussion. One participant explained that: "*There’s only five of us and we share an office so we hear everyone’s conversations, so that’s basically how we make connections ‘cause you’re just listening in on everyone else’s conversations and you pick up on things people are talking about on the phone*". Participants also communicate with provincial public health agencies with respect to potential multi-jurisdictional outbreaks, as illustrated by an example from one of the participants: "*During the course of the discussion, it came about that they had been part of a trip of about 100 people and that the majority of the people had similar symptoms but because it was an organized tour, the travel group had come from all over Canada, so in that instance, we created the exposure [in the reportable diseases database] and then I believe we… notified Public Health Ontario so that they could notify… all the other health units so that if they had a similar exposure, it needed to be linked*".

#### Health unit processes

Health unit processes can facilitate the establishment of linkages between cases. Some participants explained that health units have a point person, usually staff in a coordination or supervisory role, who would review all case details that get entered into the reportable diseases database. Through the process of reviewing the data, staff make efforts to identify links between cases. Participants often cited team meetings as a potential forum for identifying cases. Within their team meetings, discussion would be centered on risk factors for the case, potential linkages between cases, and, for example, whether a food premise was suspected to be the source of the outbreak.

Some participants also described utilizing a spreadsheet within their health unit. The spreadsheet usually contained information on cases being interviewed which included the identified pathogen, potential risk factors, onset date and other pertinent information. In addition to the spreadsheet, participants stated that epidemiologists within their health unit would retrieve case information from the reportable diseases database to identify commonalities between cases when clustering may be present. The limitation of relying on surveillance data pertained to the delay with respect to data entry by health units. As described by one participant: "*We’re four separate offices across the health unit so it’s hard for us to talk to our colleagues in the other offices, so we rely on exposures getting entered [into the reportable diseases database]…ideally within 24 hours of receiving the case*". Another participant indicated: "*I find the same problem with exposures because our exposures aren’t documented in iPHIS [Ontario’s reportable diseases database] until the case [investigation] is … closed which could take … sometimes two weeks when you’re trying to contact them to arrange interviews and trying to locate [the case] and yeah, it doesn’t prove very useful to us in putting things together*".

Participants also reported the implementation of the single interviewer approach within their health units. The single interviewer approach is defined as the administration of questionnaires on cases of interest by one or a limited number of interviewers, rather than numerous interviewers administering questionnaires. One participant described the process of using the single interviewer approach within their health unit: "*So basically what we’ve done is gone through the list of reportable enterics and divided them. We have three office locations, so my office currently has… Salmonella, E. coli, and Giardia. So if I have a Salmonella [case] and then we get another Salmonella [case], it’s going to go to one of my colleagues sitting within a few metres of me so we usually discuss our cases*". For health units not utilizing the single interviewer approach, participants emphasized that linking cases can be challenging when there are multiple interviewers conducting enteric case investigation, as illustrated by one participant: "*So it seems like we don’t have that single interviewer approach yet. We are talking about it. Apparently, it works very, very well if you have a single interviewer and in a way, you know all the cases and basically know what questions to ask, and you can link them easily. So, that’s a barrier…we are nine inspectors and each of us rotate between the cases as they come and sometimes, we do not know what the other cases are".*

### Recommendations for improving the overall process of enteric disease investigation

Focus group participants made two main recommendations for improving the overall process of enteric disease investigation. Participants expressed the need for a standardized case report form for use by all health units in Ontario in order to assist with identifying common sources of illness among health units and for managing outbreaks at the provincial and national level. In addition, participants found the focus group discussions useful for learning from others’ experiences and voiced the need for formal training and development of resource materials in regard to enteric case interviewing that would facilitate the refinement of investigation skills.

## Discussion

The focus groups allowed for the exploration of participants’ perspectives on successful interviewing techniques and the perceived barriers experienced during case investigation. As the focus groups consisted of participants from different health units with varying health unit peer group profiles, it was anticipated that the responses provided in the focus groups would vary with respect to the techniques that they used, the barriers that they experienced, and the methods used to address the identified barriers.

The study highlights the diversity of methods of case investigation practice within Ontario and may be representative of other jurisdictions as well. To overcome these various barriers, investigators used a variety of methods to communicate with cases, build rapport, identify the most likely source/potential sources of infection, provide education and link cases. A summary of the participants’ key findings are included in Table 1. In the process of meeting the legislated requirements, investigators perform a number of functions such as providing health education, collecting data for regulatory purposes ultimately to prevent further illness, enforcement, illness source attribution and outbreak detection. At times, these functions may be conflicting. It is recognized that the enforcement function could compromise the rapport required with a case in order to obtain a good history of the case’s risk factors in order to determine the most likely cause of illness for educational purposes and for identifying other cases linked by a common source. For these reasons, interviewing enteric disease cases differs from other types of interviews such as opinion polls, social science interviews, etc. that result in unique challenges faced by the enteric disease case interviewer. The different techniques and strategies required to overcome the various barriers should be incorporated in investigator training materials and health unit protocols.

**Table 1:** **A summary of the focus group participants**’ **key findings based on the study framework for an enteric disease investigation**

Topic	Key Findings
*Preparation for contacting the case*	● Have good working knowledge of the pathogen incubation period, common sources of infection, modes of transmission and control measures.
	● Review demographic information pertaining to the case such as gender, age, and residence.
*Contacting the case*	● Use voice-mail or text-messaging strategically to make contact with cases of particular demographics who do not answer the phone.
*Building rapport*	● Ensure that cases are comfortable during the interview process. Attempt to relieve any anxiety the case may have.
	● Explain the role of public health, the purpose of the interview, and that confidentiality will be maintained.
	● Allow cases to tell their story.
	● Be empathetic, and be respectful of culture and religious differences as well as language barriers.
*Identifying the source*	● Educate the case in regard to the pathogen incubation period, modes of transmission and common sources to assist with identifying the source.
	● Have the case use a calendar to remember dates, regular weekly activities, and special events close to the date of onset of symptoms.
	● Use questionnaires with common risk factors specific to the pathogen and with open and closed-ended questions to assist the case’s recall.
*Education*	● Use education to assist with identifying the source of the illness (as per above).
	● If the source of the illness is identified, education targeted at the identified source can assist with preventing further illness.
	● Be sensitive, neutral and non-judgemental when providing education pertaining to high-risk behaviours, sexual practices, and cultural practices
*Exclusion*	● Identifying a case that requires exclusion from working in a food premise, healthcare setting, or working in/attending a childcare setting can create tension in the interview. It is better to identify if exclusion is required closer to the end of the interview to avoid the loss of any rapport that was built.
*Linking cases*	● Communication with other enteric disease investigators, public health inspectors, epidemiologists, data entry clerks, and staff in other jurisdictions is useful in attempting to link cases to a common source or food premise.
	● Processes that actively attempt to link cases such as the single interviewer approach, a point person in a co-ordinator role, routine meetings of case investigators, and data analysis using spreadsheets or other software tools are useful in attempting to link cases to a common source or food premises.

Many practices identified by participants, specifically with respect to communication skills, align with the documented recommended practices outlined by a number of jurisdictions in the United States and other countries to improve enteric case investigations [9, 10, 12–15]. A majority of participants had indicated knowledge of the pathogen characteristics (i.e., common sources, incubation period, transmission routes) as the basis used to focus on the most likely sources of infection. These methods are commonly used in the identification of potential sources of infection and in the investigation of outbreaks associated with well-known risk factors. Challenges are posed, however, by outbreaks caused by novel exposures such as the 2000 and 2005 outbreaks of *Salmonella* Enteriditis associated with raw almonds and mung bean sprouts, respectively [23] [unpublished observations, Dean Middleton]. At the time, these novel sources might have been missed if investigators focused only on identifying the most likely source based on prior knowledge of pathogen characteristics. These examples speak to the importance of investigators having strong analytical abilities and an open mind to novel sources of illness. The use of open-ended questions was viewed as complementary to closed-ended questions; the open-ended questions allowing for more probing when the investigator identifies a possible cause of the illness as well as allowing for the identification of novel sources.

In addition to the two recommendations for improving the overall process of enteric disease investigation made by the focus group participants (i.e., the need for a standardized case report form as well as formal training and development of resource materials), a few recommendations can be drawn from the findings of the focus groups as a whole. The challenge of culture and language was particularly prominent in health units classified as urban centres. The ability to communicate with different ethnicities should be encouraged with investigators as the knowledge gained in cultural practices can assist in the interviewing process. Further, the single interviewer approach should be considered within health units, particularly those with large case counts, for sporadic case and outbreak investigation. A defining characteristic of the single interviewer approach is the ability for one, or a limited number, of interviewers to synthesize all information pertaining to one pathogen within a health unit. In contrast with the multiple interviewer approach, the single interviewer approach is anticipated to improve detection, and allow for a more rapid investigation, of enteric outbreaks.

### Limitations

Care must be taken in generalizing the results of this study as the responses obtained from the participants may or may not be representative of the larger population of enteric case investigators (public health inspectors and public health nurses) within Ontario or elsewhere. In particular, our study includes fewer health units classified as mainly rural that may experience unique challenges in enteric case interviewing and linking cases.

Despite efforts taken on the part of the researchers, it is possible that some participants may have been reluctant to openly express their opinion during the focus group discussions. Some participants may have adjusted what they said to conform to a popular viewpoint or for concern of offending others. Efforts made to minimize such bias included establishing focus group "ground rules" in order to create the level of comfort required to facilitate open communication.

## Conclusions

To our knowledge, this is the first study to examine the perspectives of expert enteric disease case investigators on successful interview techniques and barriers experienced during enteric case investigation. Enteric case investigators perform a number of functions when conducting a telephone interview including providing health education, collecting data for regulatory purposes, enforcement, illness source attribution and outbreak detection. Information collected must be of high quality to meet these requirements. Further, the information may be used to inform decisions about public health actions that could have significant consequences such as excluding a person from work, recalling a food item that is deemed to be a health hazard, closing a business and/or litigations. A key theme that emerged from these focus groups was the diversity of methods required in assuming the various roles. In general, the practices and techniques used by investigators align with the practices outlined in the current grey literature [8–15]. A number of recommendations, if implemented, could improve the process of enteric case investigation in the Ontario context which include; development of educational training and resources, standardized interviewing tools (i.e., case report forms), strategies to address culture and language barriers, and the implementation of the single interviewer approach. Further research is required to identify whether the various strategies presented are effective and easily implemented at an organizational level.

## Electronic supplementary material

Additional file 1: **Topic guide outlining broad, open-ended questions used to guide the focus groups.** (DOCX 32 KB)
