# Supplementary material for: A focus group study of enteric disease case investigation: successful techniques utilized and barriers experienced from the perspective of expert disease investigators
Source: BMC Public Health. 2014 Dec 18;14:1302. doi: 10.1186/1471-2458-14-1302 (PMC4301823; doi:10.1186/1471-2458-14-1302)
Supplement: Supplementary file 1 — Additional file 1: Topic guide outlining broad, open-ended questions used to guide the focus groups. (DOCX 32 KB) [file 12889_2014_7382_MOESM1_ESM.docx]

**Appendix 1**

**Enteric Disease Expert Interviewer Study**

**Focus Group Topic Guide - Questions**

1. How would you define a good or expert interviewer of enteric disease cases?
2. We defined the objectives of enteric case follow-up as:
3. Obtaining required data under the *Health Protection and Promotion Act* pertaining to the case;
4. Case and contact management to prevent transmission of illness;
5. Providing education/counselling; and
6. Attempting to identify the source (or potential sources if the definitive source is not identified) of the illness.

Do you agree with these objectives? Are there other objectives that you think should be included?

1. Establishing rapport is considered important in order to be able to obtain information from the case/client. What do you do to establish and maintain rapport? What techniques, methods or practices do you find are most successful in *establishing* and *maintaining* rapport with enteric cases?
   1. Probe: How do you get the person on the phone?
   2. Probe: How do you keep the person on the phone?
   3. Probe: Are there any barriers to establishing rapport with enteric cases while interviewing? If yes, what are these barriers? How do you try to address these barriers while interviewing?
2. The case report form or case worksheet is a tool that is used to assist with interviewing. Could you comment on the pros and cons of it when you use it during the interview process?
   1. Probe: When do you use open-ended versus closed-ended questions?
   2. Probe: Are there any barriers in regard to the use of the case report form as a tool? How do you try to address these barriers while interviewing?
3. In the context of a telephone interview, what do you do to identify the most likely source or sources of infection? What techniques, methods, or practices do you find are most successful for identifying the most likely source or sources of infection? To clarify, we are saying “most likely sources of infection” because we recognize that identifying the definitive source for one case is difficult.
   1. Are there any barriers to source identification with enteric cases? If yes, what are these barriers? How do you try to address these barriers while interviewing?
   2. Probe: Do you ask questions that are additional to the questions on the case report form to hone in on the most likely sources of infection?
   3. Probe: How important is maintaining knowledge of risk factors and emerging issues pertaining to the pathogen? If it is identified as important, how do you maintain your knowledge?
4. What do you do to link cases caused by the same source in your health unit or other health units? What techniques, methods or practices do you find are most successful for identifying links with other cases caused by the same source, or a strongly suspected source, in your health unit or other health units?
5. Probe: What successful techniques do you use in your health unit to assimilate information from a single interview to the health unit level for a given pathogen? If the investigation is done in the same office versus multiple staff interviewing in multiple branch locations?
6. Probe: What are the barriers to identifying links with other cases caused by the same source? How do you try to address these barriers?
7. If health units indicated they have regular meetings regarding investigations, please elaborate on what your team does at these meetings that enables you to make links?
8. Preventing the transmission of disease is an objective in the practice of public health. What do you do during the interview process that would attempt to prevent disease transmission? What techniques, methods or practices do you use in the interview process that would serve the objective of preventing disease transmission.
9. Prompt: Do you use case education? If yes, what successful techniques do you use?
10. Prompt: If culture-targeted education is provided, how do you become knowledgeable on cultural or regilious practices?
11. Prompt: How about case and contact exclusion from high risk settings? If yes, what successful techniques do you use to manage cases and contacts? What are the barriers?
12. Prompt: How do you follow-up on compliance to being excluded?
13. If group indicates there is no problem with compliance: Why do you think there are no barriers to getting good compliance with exclusion?
14. Is there anything that could be done in Ontario that would contribute to improving the overall process of interviewing enteric disease cases? Please consider the individual level, the health unit level, and the provincial level.
    1. Probe: How could this be implemented? In what forms?
    2. Probe: Could you comment on training? What specific aspects of training are required (i.e., what should the training cover)? How should training be delivered? Please give details.
15. Is there anything that any of you would like to share that we haven’t asked you about during this focus group? Is there a message that you would like us to take away with respect to the enteric interview process? Is there a previous question for which you would like to provide more comments?
